# Flexible Gastro-intestinal Endoscopy — Clinical Challenges and Technical Achievements

**DOI:** 10.1016/j.csbj.2017.01.004

**Published:** 2017-01-18

**Authors:** Niehls Kurniawan, Martin Keuchel

**Affiliations:** Klinik für Innere Medizin, Bethesda Krankenhaus Bergedorf, Akademisches Lehrkrankenhaus der Universität Hamburg, Glindersweg 80, 21029, Hamburg, Germany

## Abstract

Flexible gastro-intestinal (GI) endoscopy is an integral diagnostic and therapeutic tool in clinical gastroenterology. High quality standards for safety, patients' comfort, and efficiency have already been achieved. Clinical challenges and technical approaches are discussed in this short review.

Image enhanced endoscopy for further characterization of mucosal and vascular patterns includes dye-spray or virtual chromoendoscopy. For confocal laser endoscopy, endocytoscopy, and autofluorescence clinical value has not yet been finally evaluated. An extended viewing field provided by additional cameras in new endoscopes can augment detection of polyps behind folds. Attachable caps, flaps, or balloons can be used to flatten colonic folds for better visualization and stable position.

Variable stiffness endoscopes, radiation-free visualization of endoscope position, and different overtube devices help reducing painful loop formation in clinical routine. Computer assisted and super flexible self-propelled colonoscopes for painless sedation-free endoscopy need further research. Single-use devices might minimize the risk of infection transmission in the future.

Various exchangeable accessories are available for resection, dissection, tunneling, hemostasis, treatment of stenosis and closure of defects, including dedicated suturing devices. Multiple arm flexible devices controlled via robotic platforms for complex intraluminal and transmural endoscopic procedures require further improvement.

## 1 Introduction

Flexible GI endoscopy is a major diagnostic and therapeutic tool in clinical gastroenterology. Standard procedure for GI endoscopy has not changed much during the last decades. The tip of the flexible insertion tube can be bent vertically and horizontally by steering wheels via Bowden cables. Manual Insertion and retraction can be combined with rotation of the entire endoscope. Light is transmitted from the connected processor to the endoscope tip from where a chip sends back image signals from the lens to a monitor. Channels allow insufflation of the GI lumen, aspiration of fluid content, and rinsing of the lens. A larger instrumentation channel accommodates various diagnostic and therapeutic accessories. Design and cover of the endoscope allow efficient disinfection before re-use. Flexible endoscopy combines accurate mucosal visualization and therapy for the price of higher invasiveness than imaging techniques and wireless capsule endoscopy. Recommendations for technical and clinical quality standards in GI endoscopy have been established [1], [2].

Advanced optical systems for precise visual diagnosis and multiple diagnostic and therapeutic accessories which can be applied and exchanged on demand are further important features. Robotic platforms for steering of multidimensional endoscopes have already been developed.

Screening for colorectal cancer (CRC), one of the three most common types of cancer worldwide, is one of the major indications for flexible GI endoscopy [3] which outperforms other methods as fecal occult blood test, fecal DNA testing, computed tomography, magnetic resonance tomography, colon capsule endoscopy, or serum based tests [4]. Reduction in mortality by endoscopic polypectomy has been demonstrated [5]. Screening colonoscopy proofed to be safe. For example, 2.8 million community based screening colonoscopies in Germany between 2003 and 2008 had a complication rate as low 0.28%, with 0.0058% serious complications and a total procedure associated mortality of 2 patients. On the other hand approx. 26.000 carcinomas were found [6]. Nevertheless, only about 16% of the eligible population (age 55–74) participated in this screening.

Other indications for GI endoscopy are gastro-esophageal reflux disease including potentially premalignant Barrett's esophagus, gastro-duodenal ulcers, gastric cancer, treatment of small bowel bleeding, and diagnosis of inflammatory bowel disease.

Clinical tasks in flexible endoscopy are optimal characterization of lesions for targeted management, improving adenoma detection, avoiding incomplete endoscopy, reducing pain and need for sedation, infection prevention, and improvements in therapeutic endoscopy. This short review discusses clinical needs and technical solutions already achieved or under development (Table 1), addressing both engineers and clinicians.

## 2 Targeted Management of GI Lesions

### 2.1 Improved White Light Imaging

**Fig. 1:**
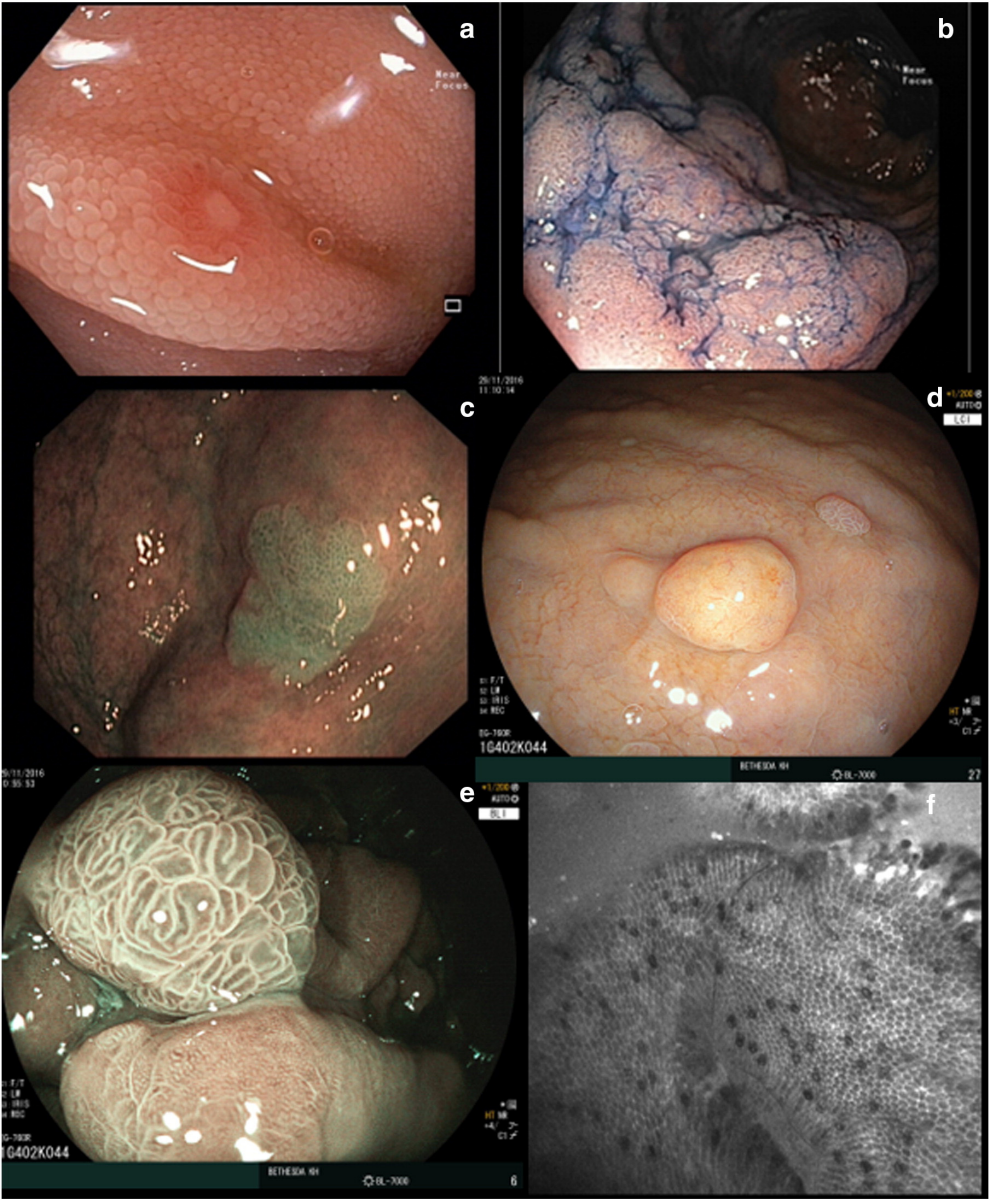
Image enhanced endoscopy (IEE): a — High definition endoscopy (HDE; small ulcer in the ileum), b — chromoendoscopy (Dysplasia associated lesion/mass DALM in ulcerative colitis), c — Narrow band imaging (NBI; sessile serrated adenoma in the colon), d — Linked Color imaging (LCI; fundic gland cyst of the stomach), e — Blue light imaging (BLI; hyperplastic gastric polyp), f — Confocal laser endoscopy (CLE; Barrett's esophagus).

Optimal quality of endoscopic images is essential for detection, classification and delineating extent of mucosal lesions. Fiber bundle endoscopy has widely been replaced by video endoscopy, and increasingly by **high definition** (HD) endoscopy (Fig. 1a). Automatic light control is a standard feature. Manual zoom and focus control have recently been introduced. With an **adjustable focus,** clear images in various distances from the endoscope may be obtained. Solid free form lens elements tuned via two piezoelectric benders for actuation have been described recently to potentially overcome technical limitations of deformable liquid lenses [7].

### 2.2 Image Enhanced Endoscopy (IEE)

IEE involves multiple techniques for improved visualization of GI lesions.

Chromoendoscopy can be performed by **dye-spray** of the mucosa with methylene blue (Fig. 1b), toluidinblue, indigocarmin for GI mucosa or Lugol for esophageal squamous cell epithelium. Although chromoendoscopy seems to improve the adenoma detection rate (ADR) it is costly and time consuming [8]. **Virtual chromoendoscopy** uses different real-time dye-less spectral color selection modes. The achieved stronger contrast improves visualization of mucosal pit pattern and vascular pattern for differentiation of adenomas and carcinoma, especially when combined with zoom and focused endoscopy. Optical filters select a narrow band width from standard full spectrum white light (WLE) in *narrow band imaging* (**NBI**, Olympus, Tokyo, Japan) (Fig. 1c) and in *compound band imaging* (**CBI**; Aohua, Shanghai, China). Other modalities like ***i-scan*** (Pentax, Tokyo, Japan), *Flexible Spectral Imaging Color Enhancement* (**FICE**, Fuji, Tokyo, Japan), and *Storz Professional Image Enhancement System* (**Spies**; Storz, Tuttlingen, Germany) involve real-time electronic post-processing algorithms for spectral color selection. By blue light imaging (**BLI**; Fuji) (Fig. 1e) narrow band color is selectively generated by the light source [9]. Linked Color Imaging (**LCI;** Fuji) (Fig. 1d) combines narrow band light from 4 LEDs to blue light or WLE illumination by post processing [10]. Classification of small colorectal polyps (≤ 5 mm) as non-neoplastic by experts using NBI may reduce the number of unnecessary polypectomies [11], [12]. However, a metaanalysis found no benefit in the ADR during screening or surveillance colonoscopy with NBI over WLE [13]. The effect of a new NBI and the CLI modes with less reduction of brightness needs further evaluation.

Different wavelength light can be used depending on the visualization requirement. **Confocal laser endoscopy** (CLE) uses blue laser light brought to direct contact with the mucosa after intravenous injection of fluorescein (Fig. 1f). This method has already been used in clinical settings detecting inflammatory and neoplastic lesions, either with dedicated endoscopes (Pentax) or with miniprobes (Cellvizio; Maunea Kea, Suwanee, GA, USA) advanced through the working channel of standard endoscopes. CLE has been used in surveillance of Barrett's esophagus, inflammatory bowel disease, differentiation of colonic polyps and other indications. However, due to high costs and missing proof of clinical benefit, further research is warranted before routine use [14].

**Endocytoscopy** (EC) allows visualization of details down the level of nuclei by contact light microscopy after staining the mucosa with methylene blue and crystal violet. The system was proposed as a miniprobe device and has also been integrated into flexible endoscopes (Olympus). A novel computer aided diagnosis system provided automatic classification of colonic polyps based on identification and characterization of nuclei during processing of EC images [15].

HD endoscopy is recommended for routine CRC screening, and real or virtual chromoendoscopy of the entire colon in high risk situations as surveillance of long standing ulcerative colitis or polyposis syndromes [16]. CLE and EC are restricted to few centers.

## 3 Improving Adenoma Detection

Up to 20–40% of adenomas are missed during standard colonoscopy [17], [18], [19]. Reasons among others can be inadequate bowel preparation [20] and short time for inspection of the mucosa during endoscope withdrawal [21]. Rinsing through integrated water channel or via the larger working channel with syringe, external water jet pump or dedicated catheter may compensate inadequate bowel preparation [22].

Approaches to detect adenomas hidden behind colonic folds by expanding the standard forward-viewing angle of 140–170° or by mechanic manipulation of folds during endoscopy are described below and summarized in Table 2.

### 3.1 Extended Viewing Field

**Fig. 2:**
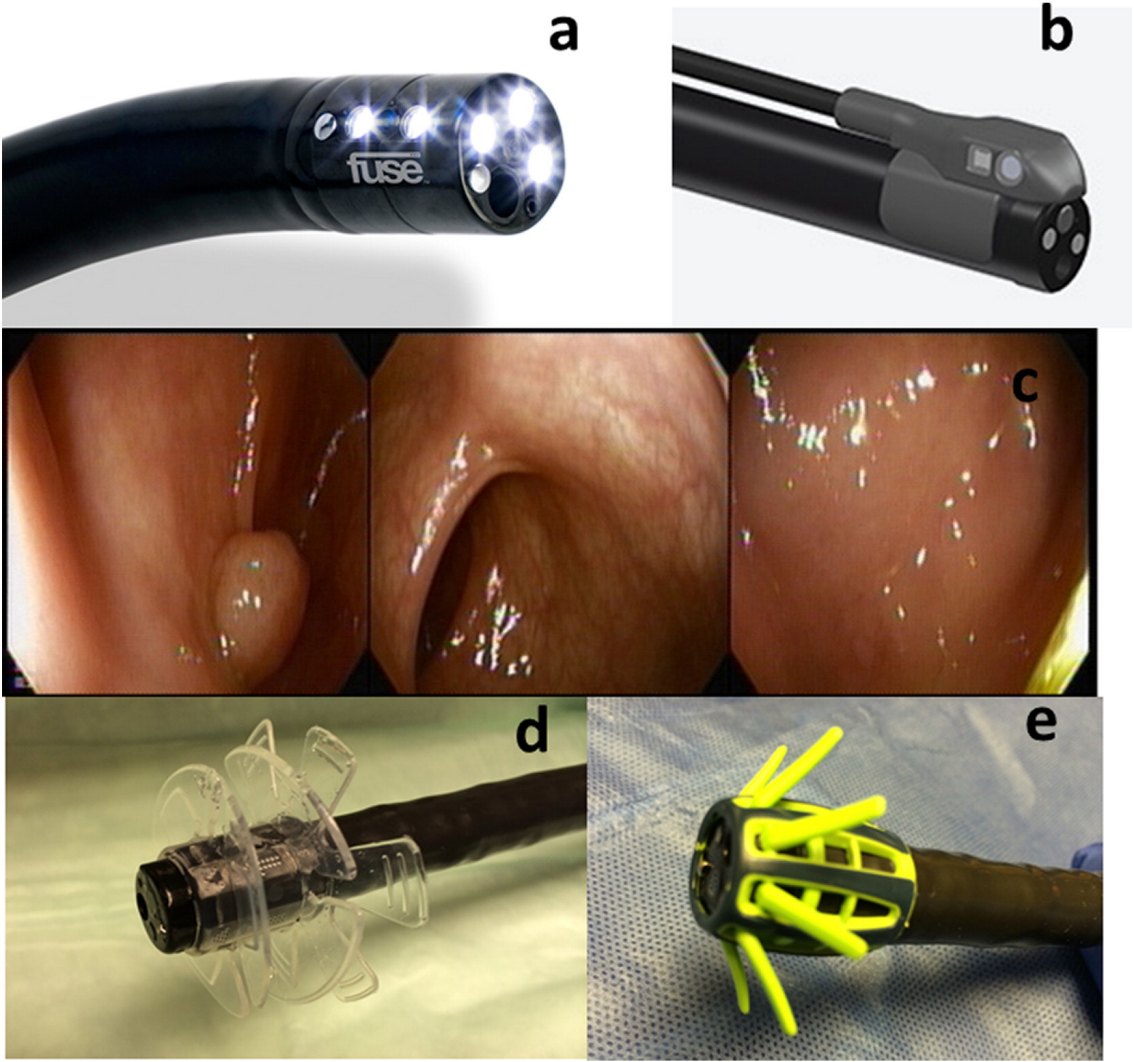
Technical approaches to increase detection of polyps behind folds. a — Distal end of Full spectrum endoscopy (FUSE, EndoChoice) endoscope, b — Third eye Panoramic (Aventis) attached to a standard colonoscope, c — FUSE monitor with 3 images. The polyp is only seen on the left monitor. Mechanical devices attached to the endoscope tip: d — EndoRings, e — Endocuff.

The ***Full Spectrum Endoscopy*** *(FUSE) colonoscopy platform* (EndoChoice, Alpharetta, GA, USA) uses a standard colonoscope with two additional cameras and light sources build into the left and right side of the distal end (Fig. 2a). The combination of three videos simultaneously shown on the monitor covers a total viewing field of 330° (Fig. 2c). In a clinical trial, *FUSE* colonoscopy detected a significantly higher number of adenomas in direct comparison to a standard colonoscopy [19].

In a similar matter, Olympus developed a prototype colonoscope with an **extra-wide angle of view** (144–232°; *EWAVE*). A single image is combined from a standard forward viewing lens and the additional convex shaped lens. A feasibility study suggested potential for a higher ADR [23].

***Omniview*** modus of the single use, self-propelled pneumatic *Aer-O-Scope* (GI View, Ramat Gan, Israel) combines a 57° forward viewing lens and a 44° lateral view circular around the entire central axis. However, in a first clinical trial there was no diagnostic benefit over standard colonoscopy [24].

The ***Third Eye Retroscope*** (Avantis, San Jose, CA, USA) is an auxiliary device with camera and light source. After insertion through the working channel of a standard colonoscope it is angulated 180° allowing an additional retrograde view of the colon with significant increase in adenoma detection of 11–23% [25], [26]. To spare the working channel the ***Third Eye Panoramic*** (Avantis) with two side viewing cameras and light sources is attached to the tip of a colonoscope (Fig. 2b). A small feasibility study reported promising results [27].

### 3.2 Flattening Folds

Another approach to increase visualization of the colonic mucosa is to straighten out folds and flexures.

***EndoRings*** (EndoAid, Caesarea, Israel) is a single use silicone rubber device attached to the distal end of the colonoscope. Flexible flaps stretch and straighten out folds during withdrawal (Fig. 2d). In a randomized trial adenomas were found in 49% with *EndoRings* compared to 29% in the standard colonoscopy group [28].

***Endocuff*** (Arc Medical, Leeds, UK) is a similar single use device using rubber arms instead of flaps to straighten out the mucosa (Fig. 2e). It was also able to increase ADR by 15% in a randomized trial [29].

The ***G-EYE balloon endoscope*** (Smart Medical Systems, Ra'anana, Israel) has a balloon permanently integrated in the distal end of a standard colonoscope. If inflated during withdrawal it straightens out folds similar to *EndoRings* or *Endocuff*. A multicenter randomized study reported an 16% increased ADR compared to a standard colonoscope [30].

Colonoscopy assisted by a transparent **cap** attached to the endoscope tip had no benefit in ADR in a large randomized trial study [31].

### 3.3 Red Flag Technology

Endoscopy with **Auto fluorescence imaging** (AFI; Olympus) has been described as a tool to highlight GI neoplasia. A metaanalysis found no increase in ADR with AFI compared to WLE [32]. Only in a subgroup of inexperienced endoscopists, ADR was significantly increased with AFI [33]. AFI has not yet proven as a reliable stand-alone red flag technique for routine application [34].

### 3.4 Feedback on Visualized Areas

To increase complete visualization of colonic mucosa, optic feedback on missed areas could be useful. For this purpose, an algorithm creating 3D images with color-coding of improperly visualized areas in a synthetic colon model based on brightness intensity analysis of endoscopic images has been proposed [35]. Furthermore, ADR may be improved by involving endoscopy nurses in the detection of polyps by additionally observing the monitor with endoscopic images [36].

Technical aids discussed in Section 3 (Table 2) have demonstrated usefulness in increasing ADR at colonoscopy, but larger studies are yet warranted. Nevertheless, physicians´ alertness and precision are still crucial for accurate diagnosis.

## 4 Avoiding Incomplete or Painful Endoscopy

Loop formation and luminal distension are main reasons for incomplete or painful colonoscopy. Technical approaches to tackle this issue are discussed below and summarized in Table 3.

### 4.1 Avoiding Loop Formation

Sedation, manual external compression, positioning of the patient and straightening and rotation of the endoscope may be useful in preventing loops and obtaining complete investigations.

Additionally, **Variable stiffness** colonoscopes (Olympus, Fuji) allow manual control of stiffness by applying tension to integrated cables. After passing a flexure with a more flexible endoscope, increasing rigidity can prevent loop formation during further advancement. The rate of complete colonoscopies was significantly higher compared to a standard colonoscope [37], [38]. Overtube or inserted rod out of thermoplastic polymers are experimental approaches for variable stiffness colonoscopes [39].

### 4.2 Position Control

The commercially available ***ScopeGuide*** (Olympus) is a positioning system emitting electromagnetic signals from special colonoscopes or inserted miniprobe to a detector beside the patient. From the signals a 3D real-time localization image of the colonoscope is displayed on a monitor resembling a virtual X-ray visualization [40]. This helps to reduce painful looping of the scope as well as localizing findings i.e. for further surgical intervention [41].

***NeoGuide*** Endoscopy System Inc. (Los Gatos, CA, USA) developed a computer assisted prototype colonoscope consisting of multiple fully articulated segments in the insertion tube. A computer algorithm calculates their position in relation to each other to generate a real-life 3D image of the insertion tube to reduce looping [42]. Additionally each segment can be remotely controlled so they can “follow” the distal end automatically around flexures [43].

### 4.3 Achieving Deep Small Bowel Endoscopy

Several **Device assisted endoscopy (DAE)** methods have been developed to reduce loops by pleating the long and hardly assessable small bowel over an overtube [44]. Repeated push and pull maneuvers are applied with inflatable balloons on the tip of the overtube (*Single balloon endoscopy*, Olympus) or on the tip of both overtube and endoscope (*Double balloon endoscopy*, Fuji) used to keep position by gripping the small bowel. A spiral endoscopy overtube (EndoEase, Spiral Medical, Bridgewater, MA, USA) translates continuous rotation into forward movement while pleating the small bowel. A new prototype endoscope with a motor driven, force controlled rotating distal spiral segment (Olympus) demonstrated feasibility in patients, allowing complete enteroscopy with a single oral approach in some of them [45], [46]. Recently, *balloon assisted enteroscopy* (Pentax/Smart Medical) with a standard colonoscope assisted by a *through the scope* (TTS) balloon has been reported [47]. After inflation of the advanced balloon catheter the endoscope is pushed forward over the catheter.

DAE methods are the gold standard for therapeutic endoscopy of the small bowel. They may also be applied in case of incomplete colonoscopy to facilitate complete inspection of the entire colon. Variable stiffness colonoscopes are widely used in routine, Scope guide is available commercially.

### 4.4 Reducing Luminal Distension

Extensive insufflation of air causes pain and hampers endoscope progression due to a larger lumen. **Insufflation of CO**_**2**_ instead of air through the scope diminishes patient's discomfort and pain during colonoscopy as it is absorbed up to 160 times faster [48], [49] and also allows deeper intubation of the small bowel during enteroscopy [50].

### 4.5 Super-Flexible and Self-Propelling Endoscopes

Another approach to reduce pain and discomfort during colonoscopy is the development of super flexible self-propelling devices. They potentially avoid necessity of sedation by smoothly following the loops without painful stretching of the bowel.

**Fig. 3:**
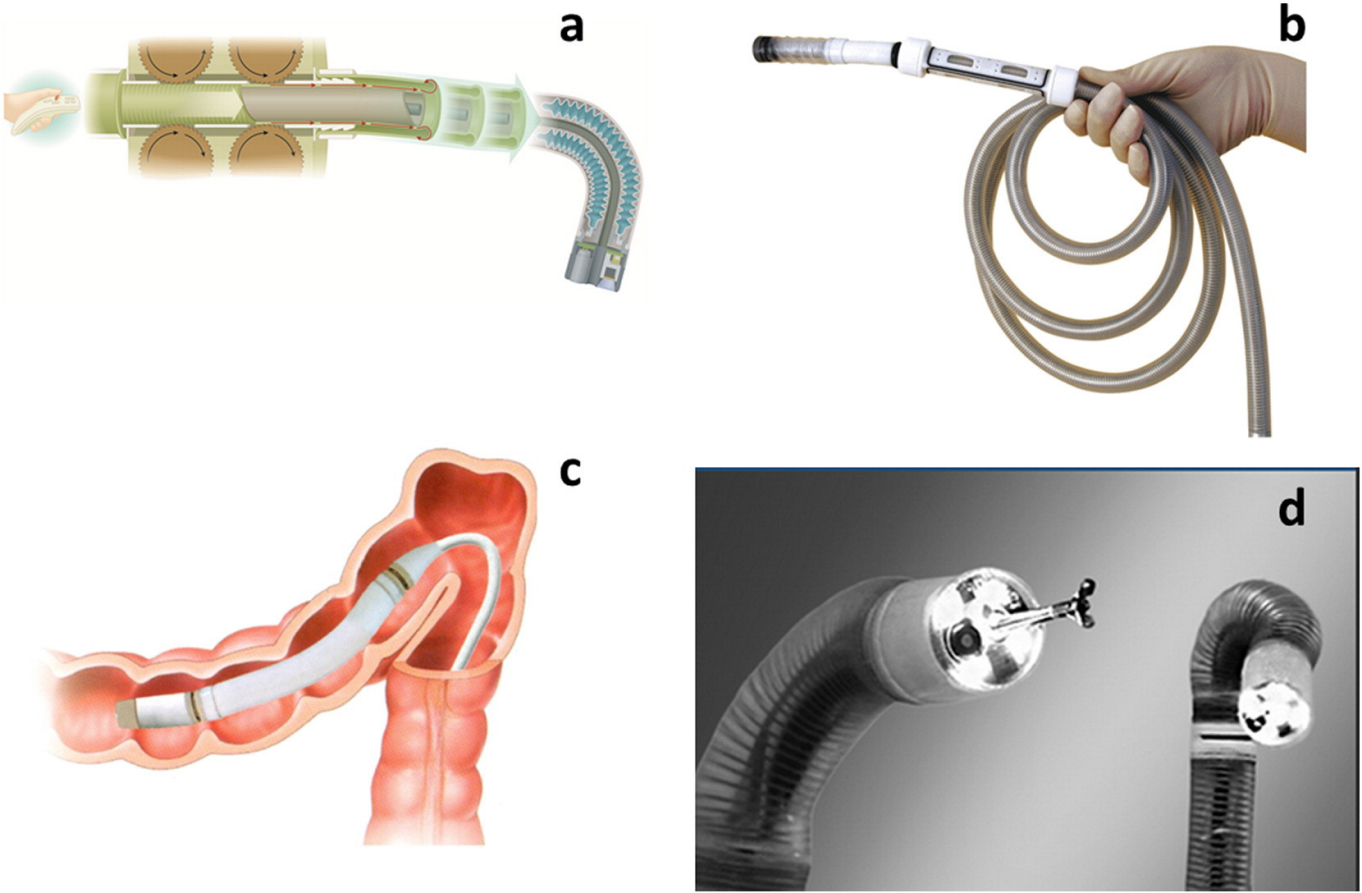
Superflexible, self-propelling colonoscopes: Invendosope C20: a — Motor roller driving unit, b — super flexible insertion tube (Invendo medical, Weinheim). ‘Inch worm’ endoscope (Endotics): c — Schematics of endoscope crawling around the splenic colon flexure, d — Endoscope tip with biopsy forceps (ERA Endoscopy, Peccioli).

The ***Invendo SC20*** (Invendo Medical, Weinheim, Germany), a single-use colonoscope including a working channel and controlled by motor rollers (Fig. 3a, b) and a handheld unit for tip control had shown excellent cecal intubation with only 5% of patients needing sedation [51]. However, this prototype has been replaced by a manually inserted single use device with standard flexibility (Invendo SC200; Section 5).

***Aer-O-Scope*** system (GI View) is a super-flexible self-propelling and self-steering endoscope tip advancing through the colon loops by controlled air pressure between inflated balloon in the rectum and at the tip of the endoscopy. Mucosa is inspected during manual withdrawal of the scope. Biopsy and polypectomy are not possible. Complete colonoscopy was possible in a clinical study in 55/56 patients without sedation [24].

The ***Endotics System*** (ERA Endoscopy, Peccioli, Italy) operates in a similar manner. A remotely controlled, single-use colonoscopy probe crawls through the colon by repeatedly adjusting its length mimicking an inch-worm (Fig. 3c). In a comparative study vs. conventional colonoscopy, *Endotics System* cecal intubation rate was 81.6% vs. 94.3% with a sedation rate of 0% vs. 19.7% [52]. Recently a working channel for biopsy and polypectomy was added (Fig. 3d).

These super flexible endoscopes aiming at painless sedation free colonoscopy are either not on the market or not widely used in routine yet. Hence it is not clear if a higher rate of complete colonoscopies than with standard flexible endoscopes could be achieved in the future.

## 5 Infection Prevention

**Fig. 4:**
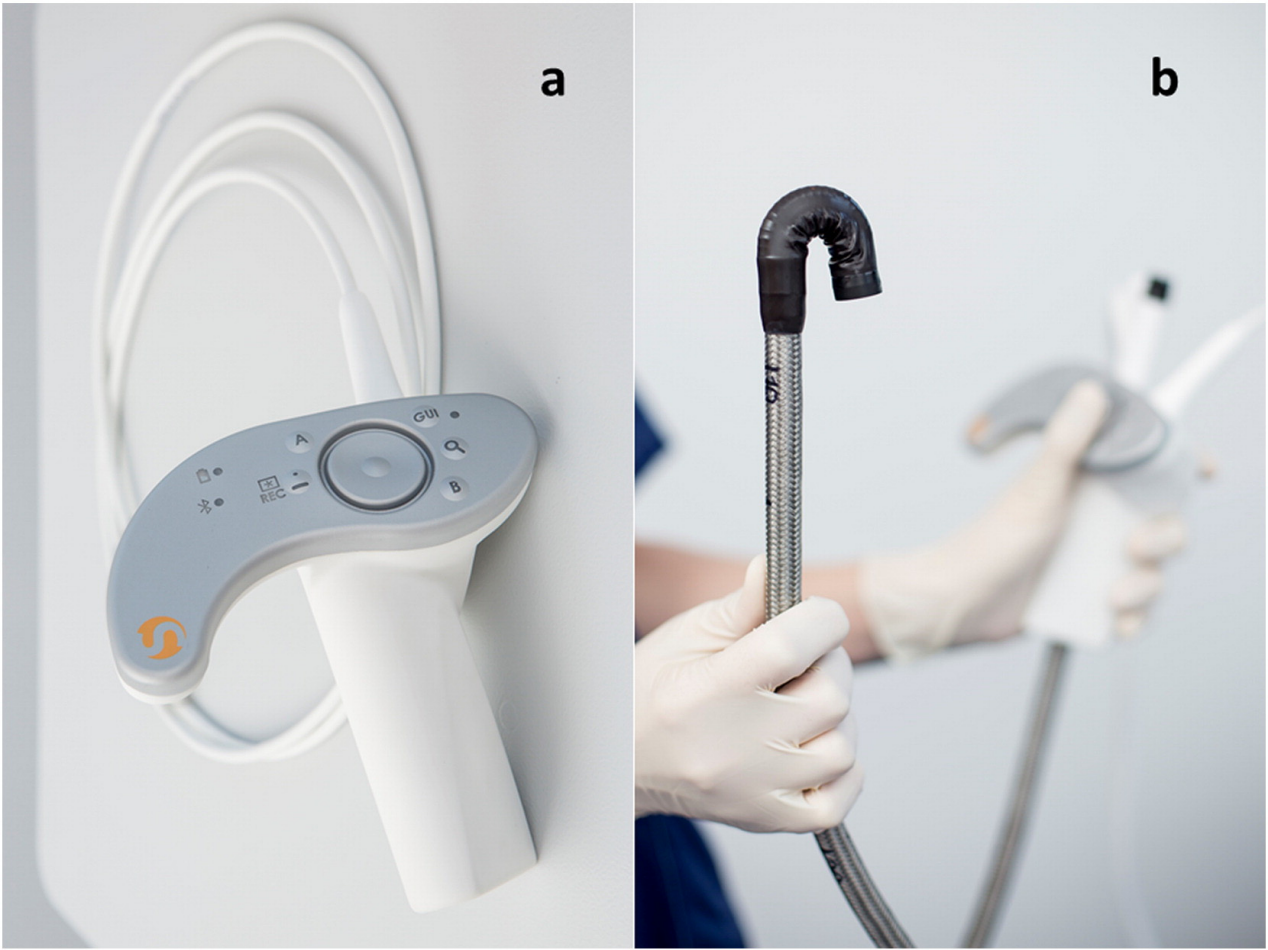
Single use endoscope. Invendo SC200: a — Hand held unit for electronically controlled tip angulation, b — single use insertion tube (Invendo medical, Weinheim).

Although high standards for processing and disinfection of endoscopes have been established there are still issues related to bacterial outbreaks. Especially the working channel may pose a challenge [53]. Wireless capsule endoscopes produced for single use omit the risk of transmitting infections but presently are only diagnostic. Furthermore, the new super-flexible endoscopes (*Aer-O-Scope*, *Endotics*, and *Invendo* [Fig. 4]) are single use devices.

With the *ColonoSight* (Stryker GI, Haifa, Israel) a hybrid solution had been developed. The reusable colonoscope does not need disinfection as a single-use cover including the working channel prevents the endoscope from contact with potentially infectious agent. No bacterial contamination of endoscope was found [54].

However, none of these single use flexible endoscopes has entered clinical routine yet.

## 6 Therapeutic Endoscopy

Flexible endoscopy is indispensable for treatment of GI lesions. The therapeutic spectrum has increasingly been extended, during routine procedures as well as by referrals to complex interventions potentially avoiding surgery. Through the scope (TTS), over the scope (OTS) and over the wire (OTW) accessories are available for various purposes.

### 6.1 Endoscopic Resection

**Fig. 5:**
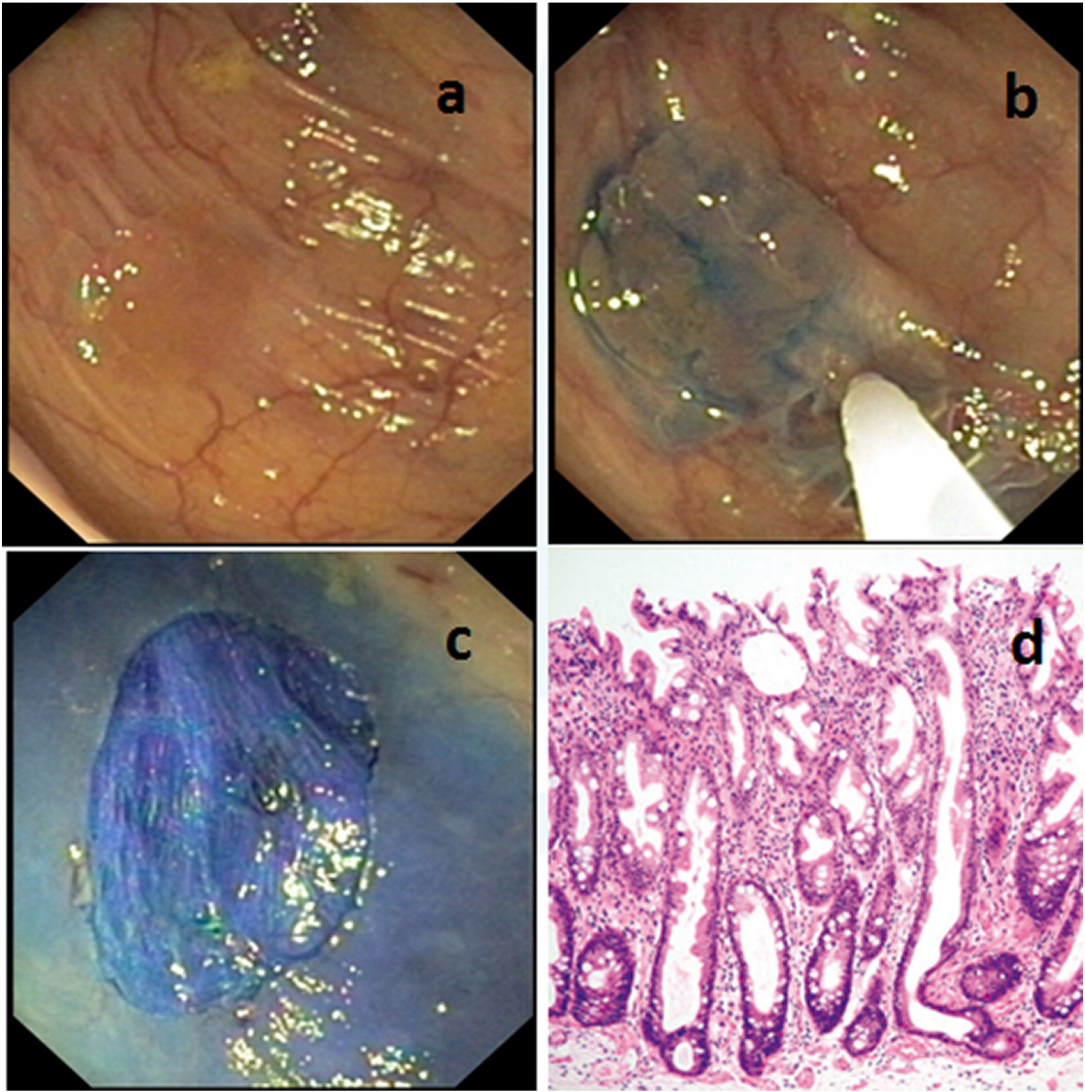
Endoscopic diagnosis and treatment in the same session, sessile serrated adenoma: a — The flat lesion is hardly visible with standard WLE. b — Demarcation after injection with methylene blue. c — Result after mucosectomy. d — Histology (courtesy of Prof. Sören Schröder) showing proliferation of crypts at the base of the specimen.

**Polypectomy** of adenomatous polyps detected at colonoscopy is integral part of screening procedures. Standard TTS instruments are biopsy forceps for histology including complete removal of lesions up to 5 mm, and polypectomy snares with electrocautery for larger polyps. Flat and laterally spreading adenomas can be lifted by submucosal injection of saline before **endoscopic mucosal resection (EMR)** with a snare. Stain added to the saline may be useful for better delineation of the margins. Larger lesions have to be resected in parts (piece meal). Although IEE can correctly classify most lesions accurate, histology is still important for correct classification (Fig. 5) including detection of malignant areas that might have developed within adenomatous polyps.

Superficial malignant lesions of the mucosa can be resected en-bloc by **endoscopic submucosal dissection (ESD)**. After injection of fluid into the submucosal space and circular incision of the mucosa around the lesion, the submucosa is dissected underneath the lesion using various types of TTS knifes for incision and dissection, needles for repeated injection, and graspers for coagulation of vessels [55]. Instruments combining injection and dissection avoid the need for frequent exchange. By ESD, even large lesions can be treated endoscopically in specialized centers. However, endoscopic treatment is limited biologically by increasing risk of local lymph node metastasis with deeper infiltration requiring radical oncologic surgery for curative treatment. Detailed recommendations on appropriate use of EMR, ESD or surgical resection depend on presence of malignancy, localization, size, depth of invasion, and differentiation of GI neoplasia [56].

### 6.2 Closure of GI Wall Defects

**Fig. 6:**
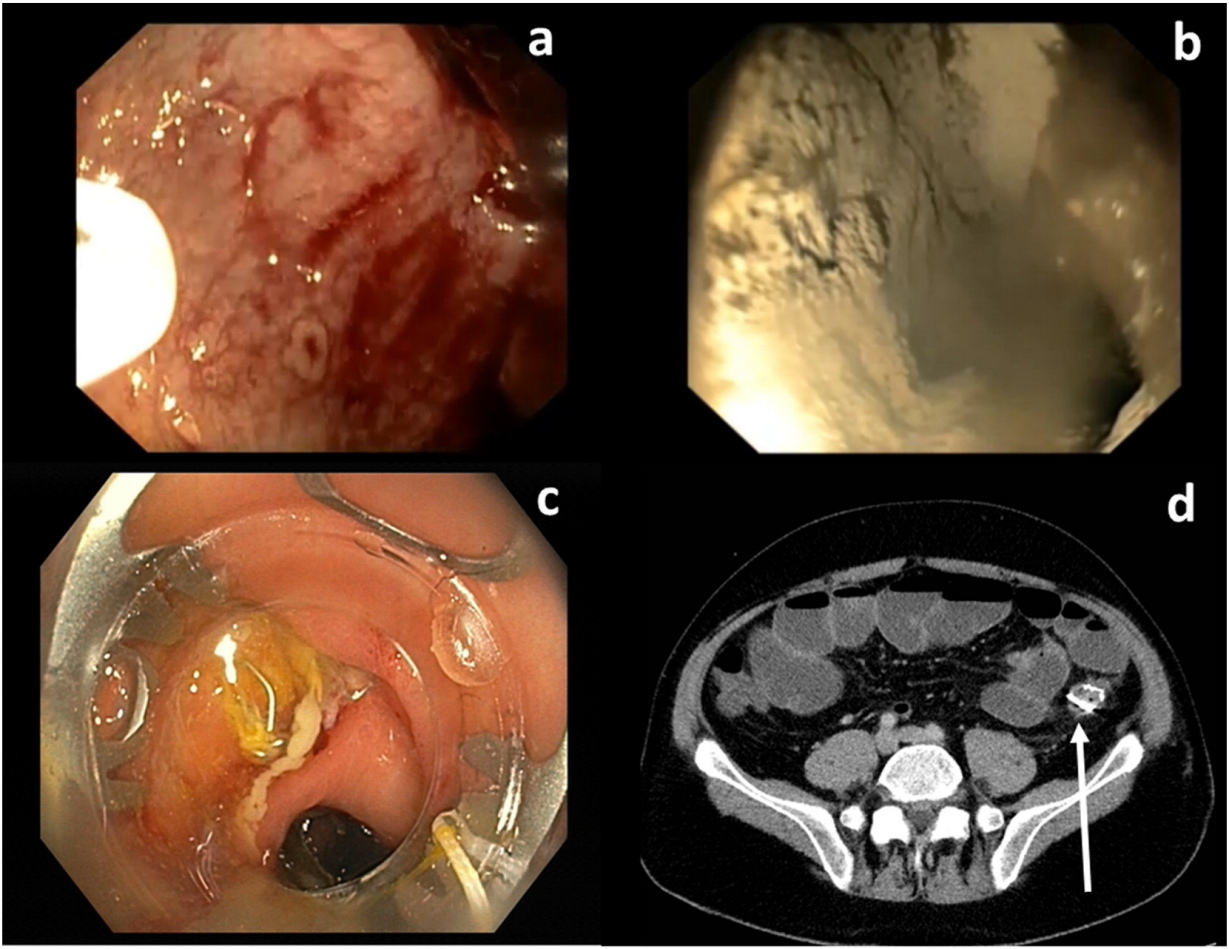
Endoscopic therapy. Through the scope (TTS): a — Duodenal bleeding, Hemospray catheter. b — successful hemostasis after application of hemospray powder. Over the scope clip (OTSC): c — endoscopic view of a postoperative fistula in the colon, OTSC device on the tip of a standard colonoscope. d — Computed tomography scan with OTSC in situ after successful closure of the fistula.

Small perforations of the GI wall including those after endoscopic resection or after surgery can be treated endoscopically in selected patients. Multiple TTS metallic clips are useful to adapt wound margins. Larger defects can be closed with a TTS loop used to adapt several clips at both sides of the defect. Alternatively, a larger clip mounted over the scope clip (OTSC) may close margins sucked into the cap of the device (Fig. 6c, d). A full thickness resection device (FTRD) is a modification allowing to suck the lesion with the entire GI wall into a cap and to place an OTSC before full thickness resection [57]. Recently, successful closure of complete GI wall defects [58] and fistulae [59] with a suturing device attached to an endoscope (Overstitch, Apollo, Austin, TX, USA) has been reported.

### 6.3 Treatment of Stenosis

GI strictures can often be dilated with hydrostatic balloons advanced through the scope. Bougies are advanced over a wire placed through the endoscope after withdrawal of the scope, as well as larger pneumatic dilation balloons or self-expanding metal stents [60]. However, in malignant strictures endoscopy can only provide palliation or bridging before definitive therapy.

Recently, PerOral Endoscopic Myotomy (**POEM**) has been introduced as alternative to surgery of hypertrophic lower esophageal sphincter in achalasia causing dysphagia. The esophageal submucosa is dissected after incision of the superficial mucosa to create a tunnel allowing advancing the endoscope distally for incision of the deep muscular layer of the hypertrophic sphincter. Finally, the mucosal defect is closed by clips [61]. The method has further been applied for similar incision of the pyloric sphincter in refractory gastroparesis, starting the tunneling in the distal stomach [62].

### 6.4 Endoscopic Hemostasis

Endoscopic treatment is the first choice in GI bleeding from ulcer or vascular lesions. TTS needles for injection of saline, epinephrine, or tissue glue; hemoclips for mechanic hemostasis; coagulation probes for destruction of vascular lesions, and catheters for hemostatic sprays (Fig. 6a, b) have expanded the therapeutic armamentarium. Larger OTS devices are available as rubber band ligation for routine treatment of varices and OTS clip as rescue therapy for bleeding ulcers.

### 6.5 Metabolic Endoscopy

Endoscopy is used for a long time to guide percutaneous placement of feeding tubes in patients with jeopardized nutrition. However, nowadays, obesity and diabetes are increasing challenges. Hence, similar tubes have even been used to empty the stomach in obese patients. Endoscopically placed gastric balloons reduce the gastric lumen temporarily. Permanent gastroplasty is typically performed surgically but has also been performed endoscopically by stapling devices as experimental articulating circular endoscopic (**ACE**) system [63] and **Transoral Gastroplasty** (TOGA, Satiety, Palo Alto, CA, USA) [64] or by suturing devices as **Overstitch** [65], and **Endomina**, (EndoTools SA, Gosselies, Belgium) [66].

The endoscopically implanted duodeno-jejunal bypass sleeve **Endobarrier** (GI Dynamics, Bosten, MA, USA) improved obesity and diabetes, but proximal fixation hooks in the duodenum may cause adverse events [67].

### 6.6 Multi-Tasking Endoscopic Platforms

Complex endoscopic resection of large or unfavorably positioned lesions is augmented by a stable endoscope position. A robotic platform (University of Twente, Netherlands) additionally uses an intuitive interface to control motorized endoscope movements and deflection of the tip via joystick or touch pad [71]. Robotic steering proofed more effective than standard handling in a simulation model [68], but first clinical test demand further improvement and training [69]. Additionally, haptic feedback of forces measured during insertion and rotation of the endoscope by a slave robot could be reflected successfully to the steering handle in the master unit of Endoscopic operation robot ver.3 [70]. With flexible multidimensional remote controlled endoscopes transmitting force via Bowden cables instead of using traditional rigid laparoscopic instruments non-linear force transmission with backlash hysteresis have to be compensated during remote control [71].

A double channel endoscope (R-Scope, Olympus) allows limited triangulation with an additional grasper. Procedure times for ESD could be shortened in a Japanese series [72] while initial Western experience was less favorable [73].

**Natural Orifice Transluminal Endoscopic Surgery (NOTES)** aims to reduce invasiveness of traditional transabdominal surgery by accessing the peritoneal cavity through natural lumina as stomach, colon or vagina [74]. Sufficient insufflation of the peritoneal cavity with CO_2_ requires higher flow rates than provided by standard endoscopes for distension of the GI lumen. Adopting high flow devices with pressure control from laparoscopy could be helpful [75]. Multiple arm flexible endoscopic devices and robotic platforms provide multiple degrees of freedom to enable surgical principle of tissue retraction and triangulation [76]. Although developed for transluminal approach these platforms may be used for intraluminal procedures as well.

**Fig. 7:**
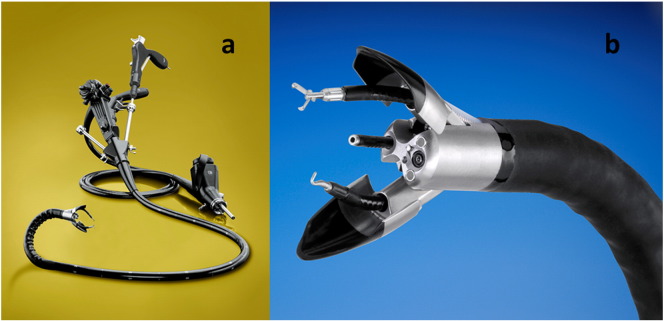
Multi-dimensional flexible endoscope. a — Anusbisope, b — close-up view of the endoscope tip with two flexible instrumentation arms (Storz, Tuttlingen).

***Anubiscope***™ (Storz) [77] with two endoscopic arms of the flexible instrument allowed triangulation of the tissue also during endoluminal endoscopic submucosal dissection (ESD) with complete and safe resection (Fig. 7) [78].

The ***EndoSamurai*** (Olympus) robotic platform translates bimanual actions into movements of the two flexible small caliber instruments in a flexible endoscope. This system outperformed a conventional dual channel flexible endoscope [79] and reached accuracy of traditional laparoscopic instrumentation in a bio model, although procedure times were longer [80].

***Cobra*** and ***TransPort*** flexible endoscopy systems (USGI, San Clemente, CA, USA) provide a platform for transluminal endoscopy. The *TransPort* device with 4 working channels allows simultaneous application of flexible endoscopes and surgical instruments [81].

***Master And Slave Transluminal Endoscopic Robot*** (**MASTER)** platform translates bimanual steering to a flexible endoscope with two instrumentation arms with nine degrees of freedom [82]. Feasibility for ESD in vivo [83] and ex-vivo [84] pig models and even in patients [85] has been shown.

***Direct drive endoscopic system*** (DDES) (Boston Scientific, Natick, MA, USA) allows to bimanually directing two instruments in an endoscopic sleeve via a robotic platform by one operator [86], [87]. Optics is separated from instruments by adding a small caliber flexible endoscope.

The principle of a semi flexible laparoscope creating 3D images from two separate cameras (**Endoeye Flex 3D,** Olympus) [88] might be adopted in future for flexible GI endoscopes especially in multidimensional platforms.

Most cases of NOTES have been performed as hybrid transvaginal cholecystectomy still with limited transabdominal augmentation. Transgastric and transcolonic access have also been used but are not yet ready for use in clinical routine [89].

## 7 Future Perspectives

Wireless technique of capsule endoscopy, the first line diagnostic tool for the small bowel is already available for diagnostic endoscopy of the upper and lower GI tract. However, detailed characterization of lesions including histology and therapy will require flexible endoscopy as gold standard for the next years. An endoscope combining possible advantages would be a single-use, super flexible, self-propelled device for a pain free procedure without sedation. Optional increase in rigidity could provide a stable position for therapy. Ideally, a small caliber endoscope provides ample working channels with sufficient size to easily apply and exchange all appropriate accessories. Additionally, real-time image processing programs could assist in lesion detection and characterization. Dedicated robotic platforms might further augment intraluminal and transmural complex therapies augmented by 3D imaging.

## Figures and Tables

**Table 1:** Clinical challenges in flexible endoscopy and technical approaches to tackle them.

Clinical challenge	Technical challenge	Principle	Method
Optimal characterization of lesions for targeted management	Improved white light imaging	Improved image resolution	High definition endoscopy
	Image enhanced endoscopy	Chromoendoscopy	Dye spray
		Virtual chromoendoscopy	Spectral light selection
		Virtual biopsy	Confocal laser endoscopy, endocytoscopy
Improving adenoma detection	Extension of viewing field	Additional integrated cameras	Full Spectrum Endoscopy (FUSE), Extra wide angle of view endoscopy (EWAVE), Omniview
		Additional attached cameras	Third eye (retroscope/panoramic)
	Visualization of mucosa behind folds	Flattening bowel folds	Attached flaps, integrated balloon, Cap
	Red flag technologies	High lightening neoplasias	Autofluorescence
	Feedback on visualized areas	3D reconstruction software	Brightness intensity analysis
Avoiding incomplete endoscopy	Avoiding loop formation	Variable stiffness colonoscopes	Variable tension of cables
			Thermoplastic rod or overtube
	Position control	Visualization of scope position	Scope Guide, Neo Guide
		Computer assisted memory function of scope segments	Neo Guide
	Achieving deep small bowel intubation	Pleating of the bowel	Device assisted endoscopy (Single, double or assisting balloon, spiral)
Reducing pain/need for sedation	Reducing luminal distension	Improved resorption of insufflated gas	Insufflation of CO_2_
	Super-flexible self-propelling scopes	Propelled by air pressure	Aer-O-Scope, ColonoSight
		Propelled by motor rollers	Invendo SC20
		Inch worm technique	Endotics
Infection prevention	Single use devices	Single use endoscope	*Aer-O-Scope*, *Endotics*, *Invendo* SC20/E200
		Single use sheath	ColonoSight
Therapeutic endoscopy	Resection of premalignant lesions	Biopsy	Biopsy forceps
		Polypectomy	Polypectomy snares
		Endoscopic mucosa resection (EMR)	Injection needles, snare, clips
		Endoscopic submucosal dissection (ESD)	Dissection knifes, coagulation graspers
		Endoscopic full thickness resection	Full thickness resection device (FTRD)
	Triangulation	Dual channel endoscope	R-Scope
	Stabilization of scope position	Motor roller driven	Robotic platform
	Treatment of stenosis	Dilatation	Balloon dilation
			Bougienage
		Obtaining passage	Stent placement
		Myotomy in achalasia or refractory gastroparesis	PerOral Endoscopic Myotomy
	Closure of GI wall defects	Metall clip	Through the scope clip
			Over the scope clip (OTSC)
		Suture	Endoscopic hand suturing
	Hemostasis in GI bleeding	Metall Clip	Through the scope clip
			OTSC
		Coagulation	Electro, Argon-Plasma, Heaterprobe
		Cohesive and adhesive compound	Hemospray, EndoClot
		Obliteration of varices	Rubber band ligation
			Injection of Histoacryl glue
	Endoscopic treatment of obesity	Gastroplication	Endomina, articulating endoscopic stapler, Transoral Gastroplasty (TOGA)
		Duodeno-jejunal sleeve	Endobarrier
	Natural Orifice Transluminal Endoscopic Surgery	Multidimensional robotic platforms	Anubiscope, EndoSamurai, Cobra, TransPort, Master And Slave Transluminal Endoscopic Robot (MASTER), Direct drive endoscopic system (DDES)

**Table 2:** Endoscopes and endoscopic devices developed to increase visualization of the mucosa and consecutively adenoma detection rate.

Device	Patients	Comparative method	Results device (vs. standard colonoscopy)	Author
*Full Spectrum Endoscopy (FUSE)*	185	Device and standard colonoscopy, randomized order	7% vs. 41% Adenoma miss rate (AMR) (p < 0.0001) 34% increased Adenoma detection rate (ADR)	Gralnek et al. [19]
*Extra wide angle view (EWAVE)*	47	None	Proof of feasibility	Uraoka et al. [23]
*Omni View* (Aer-O-Scope)	56	Device followed by standard colonoscopy	12.5% polyp miss rate	Gluck et al. [24]
*Third Eye Retroscope*	349	Device and standard colonoscopy, randomized order	22.6% vs. 45.8% AMR 23.2% increased ADR	Siersema et al. [25]
*Third Eye Panoramic*	33	None	Proof of feasibility ADR 45%	Rubin et al. [27]
*EndoCuff*	492	Device and standard colonoscopy, randomized	14.7% increased ADR (p < 0.0001)	Floer et al. [29]
*EndoRings*	116	Device and standard colonoscopy, randomized order	10.4% vs. 48.3% AMR (p < 0.001) 20.3% increased ADR (p = 0.025)	Dik et al. [28]
*G-EYE balloon endoscope*	106	Device and standard colonoscopy, randomized order	7.5% vs. 44.7% AMR (p = 0.0002) 14.5% increased ADR (p = 0.115)	Halpern et al. [30]
Cap-assisted colonoscopy	1113	Device or standard colonoscopy, randomized	42% vs. 40% ADR (p = 0.452)	Pohl et al. [31]

**Table 3:** Endoscopes and endoscopic devices developed to reduce loop formation.

Device	Single use	Working channel	Patients	Comparative method	Results device (vs. standard colonoscopy)	Author
*Variable stiffness colonoscope*	No	Yes	1923	Device or standard colonoscopy, Metaanalysis	Cecal intubation rate higher vs. standard (OR = 2.08, 95% CI: 1.29–3.36)	Othman et al. [38]
*Scope guide*	n/a	n/a	233	Device (n = 133) or standard colonoscopy (n = 100)	93.9% vs. 95% cecal intubation rate 6.8 min vs. 6.5 min to cecum	Wehrmann et al. [40]
*Neo guide*	No	Yes	11	None	100% cecal intubation rate	Eickhoff et al. [43]
*Invendo SC20*	Yes	Yes	61	None	98.4% cecal intubation rate 15 min to cecum 4.9% sedation	Groth et al. [51]
*Aer-O-scope*	Yes	No	56	Device followed by standard colonoscopy	98% cecal intubation without sedation after 13.3 + 7.6 min	Gluck et al. [24]
*Endotics system*	Yes	Yes	71	Device followed by standard colonoscopy	82% vs. 94% cecal intubation rate (p = 0.03) 45.1 min vs. 23.7 min procedural time (p < 0.0001) 0% vs. 14% Sedation (p < 0.0001)	Tumino et al. [52]
*Sightline ColonoSight*	Yes (partially)	Yes	178	None	90% cecal intubation rate 11.2 min to cecum	Shike et al. [54]
